# Can general practitioners do the follow-ups after surgery with ventilation tubes in the tympanic membrane? Two years audiological data

**DOI:** 10.1186/1472-6815-14-2

**Published:** 2014-04-05

**Authors:** Bjarne Austad, Irene Hetlevik, Vegard Bugten, Siri Wennberg, Anita Helene Olsen, Anne-Sofie Helvik

**Affiliations:** 1 General Practice Research Unit, Department of Public Health and General Practice, Norwegian University of Science and Technology (NTNU), PO Box 8905, 7491 Trondheim, Norway; 2 Sjøsiden Medical Centre, Trondheim, Norway; 3 Department of Neuromedicine, Faculty of Medicine, NTNU, Trondheim, Norway; 4 Department of Ear, Nose and Throat, Head and Neck Surgery, St. Olavs Hospital, Trondheim, Norway

**Keywords:** Otitis media, Tympanostomy tubes, Follow-up care, General practice, Implementation, Clinical guidelines, Hearing, Children

## Abstract

**Background:**

A university hospital in Mid-Norway has modified their guidelines for follow-up after insertion of ventilation tubes (VTs) in the tympanic membrane, transferring the controls of the healthiest children to general practitioners (GPs). The aim of this study was to evaluate the implementation of these guidelines by exploring audiological outcome and subjective hearing complaints two years after surgery, assessing if follow-ups in general practice resulted in poorer outcome.

**Methods:**

A retrospective observational study was performed at the university hospital and in general practice in Mid-Norway. Children below 18 years who underwent surgery with VTs between Nov 1st 2007 and Dec 31st 2008 (n = 136) were invited to participate. Pure tone audiometry, speech audiometry and tympanometry were measured. A self-report questionnaire assessed subjective hearing, ear complaints and the location of follow-ups. This study includes enough patients to observe group differences in mean threshold (0.5–1–2–4 kHz) of 9 dB or more.

**Results:**

There were no preoperative differences in audiometry or tympanometry between the children scheduled for follow-ups by GPs (n = 23) or otolaryngologists (n = 50). Two years after surgery there were no differences between the GP and otolaryngologist groups in improvement of mean hearing thresholds (12.8 vs 12.6 dB, p = 0.9) or reduction of middle ears with effusion (78.0 vs 75.0%, p = 0.9). We found no differences between the groups in terms of parental reports of child hearing or ear complaints.

**Conclusions:**

Implementation of new clinical guidelines for follow-ups after insertion of VTs did not negatively affect audiological outcomes or subjective hearing complaints two years after surgery.

## Background

A large number of children with otitis media with effusion or recurrent otitis media undergo surgery with ventilation tubes (VTs) placed in the tympanic membrane, also known as tympanostomy tubes or grommets. This is done to improve hearing and speech development and to reduce ear complaints [1]. It is described as the most common ambulatory surgery performed on children in the United States [2]. In a cross-sectional questionnaire study of 40,000 Norwegians, the estimated life-time prevalence of surgery was about 12% [3].

The long-term results of VTs are discussed in the literature [4,5]. A Cochrane report from 2010 concluded that they had a small effect on the hearing threshold for children with otitis media with effusion, but this effect diminishes after six to nine months [6]. For recurrent acute otitis media a systematic review found VTs to reduce only one attack of acute otitis media the first six months after surgery [7]. Still, once surgery has been performed, “follow-up care is required to assure that the tubes are functional, hearing loss has been corrected, and potential complications are properly diagnosed and managed” [8]. Examples of complications are otorhea, occlusion of tubes, premature extrusion, persistent perforation, tympanosclerosis, focal atrophy of the tympanic membrane, retraction pocket and cholesteatoma [9].

### Clinical guidelines

Guidelines regarding follow-up care give different advices concerning when, how and by whom the controls should be made [10-12]. The American Academy of Otolaryngology - Head and Neck Surgery recommend the initial control within one month after tube placement, then at least once every six months until the tubes extrude [13]. The Norwegian national guidelines are similar with the first control one month after surgery, but then once every four months until the results are as good as possible [14]. A study from Scotland documented however that the majority of the outpatient clinic controls resulted in no clinical interventions, and therefore questioned the need for regular follow-ups. They suggested one control at three months, and then only further controls for children with impaired hearing or complications [15]. The Swedish Council on Health Technology Assessment completed a systematic literature review focusing on the documentation of VT treatment. They could not conclude how and when children with inserted VTs best ought to be followed up [16]. Follow-ups of VTs are mostly done by otolaryngologists, and partly by pediatricians, i.e. on a more expensive health care level than general practice [10,17]. Because of the great number of children with VTs, this may be a burden for the specialist health care service and also imply reduced cost-effectiveness for the overall healthcare system.

### Change of guideline

In 2007 a university hospital in Mid-Norway modified their guidelines for follow-up care after VT surgery in agreement with the general practitioners (GPs) in the municipality. Previously, all children had follow-ups at the outpatient clinic. After the guideline modification children with normal hearing or minor hearing loss should have follow-ups in general practice; first at six months and again at 18 months after surgery. Children with medical syndromes, hearing loss above 30 dB in at least one frequency (0.5–1–2–4 kHz) in the worst ear or unresolved hearing (not audiological tested, but with suspected hearing loss), were recommended to continue their follow-ups at the outpatient clinic. Point of time for control at the outpatient clinic could vary depending on the severity of the disease. The GPs received a simple guideline on how to handle complications in relation to VT treatment, such as to treat a plugged tube with ear drops for two weeks followed by another control by the GP and also to refer back if a VT was not rejected within 18 months [18]. The parents were informed verbally and in writing about the new procedure and instructed to make the appointments with their GP themselves [19].

### Implementation

Development and implementation of clinical guidelines are regarded to be among the major strategies for knowledge transfer [20]. Therefore it is important to understand how the implementation process works, identify barriers against implementation [21-23] and to analyze the outcome after the guideline has been changed [24]. Lack of adherence to guidelines is well known, both in relation to process [25,26] and outcome [27] and will necessarily have the consequence that desired effects fail to appear [28-30]. Implementation research has revealed that multifaceted methods for guideline implementation are more successful than use of single methods [31,32]. As a consequence, multifaceted strategies were used for implementation in this study, both at the hospital and in general practice. We have in another paper described the process of implementation [33]. The hospital adhered to the guidelines in two-thirds of the patients; delegating more patients to primary care than the guidelines recommended. The implementation was successful when it came to patients consulting their GP for controls; all but one (95.7%) went to control the VTs.

This paper examined the outcome, i.e. the audiological outcome and subjective hearing complaints two years after insertion of VTs. We focused on whether the implementation of new clinical guidelines, allowing GPs to control the VTs in one group of children, negatively affected hearing thresholds, degree of speech recognition, or middle ear function for the children.

## Methods

Inclusion criteria were insertion of a VT in at least one ear in patients below 18 years at a university hospital in Mid-Norway within the first 14 months after the guidelines were modified; i.e. between Nov 1st 2007 and Dec 31st 2008. During this period 137 children underwent surgery. One child was excluded because of a co-existing severe disease, so 136 were eligible for the study.

**Figure 1:**
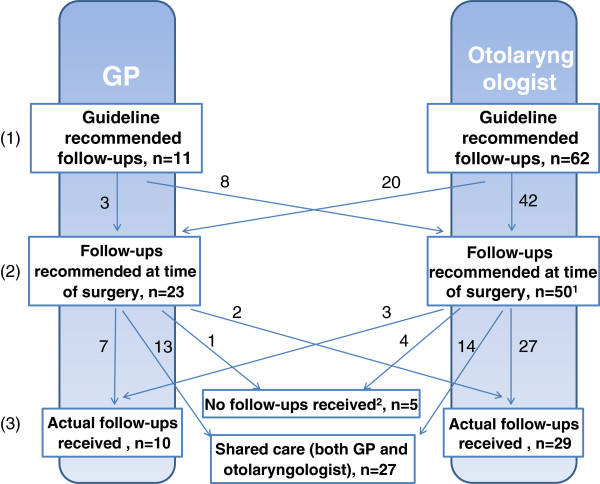
**Localization of follow-ups for the participants (n = 73) according to: (1) the guidelines, (2) the recommendations at time of surgery, and (3) the actual control. **^1^Missing data from two participants ^2^Reasons for no follow-ups are explored in a previous paper [33].

Close to two years after surgery (24 ± 3 months) all 136 children with parents/guardians were invited by letter to participate in this study. The invitation included an appointment for an audiological consultation and a questionnaire. The parents and children completed the questionnaire latest at the time of consultation. After completing the audiological examination, participants with severe medical syndromes were excluded from the analysis in this paper to make the groups followed up by GPs and otolaryngologists easier to compare. The allocation of follow-ups after surgery was not randomized, but was made by the otolaryngologist at the hospital who inserted the VTs. The decision was based on the guidelines and clinical judgment. The scheduled follow-ups were not always in concordance with the guideline recommendations or where the children actually had their controls for different reasons [33]. Figure 1 contains a flowchart of localization of follow-ups.

The participants were included after informed written consent. Due to Norwegian regulations parents/guardians had to give consent on their own behalf and on behalf of children under the age of 16. Adolescents 16 years and older consented on behalf of themselves. The study was approved by the Regional Ethics Committee in Sør-Trøndelag (2009/155-2) and the Norwegian Social Science Data Service (NSD).

### Audiological testing before and 24 ± 3 months after surgery

Information about the audiological tests prior to surgery was obtained from the medical record of the participants. The testing after surgery was committed at the hospital by two experienced audiologists in a soundproof room. Cerumen was removed prior to examination. Based on the recorded findings, the children with need were offered a medical examination with an otolaryngologist within a few days.

The audiological measures consisted of a pure tone audiogram, speech recognition tests and tympanometry. In cases where the child due to age or other reasons could not cooperate in these investigations, play audiometry or informal hearing tests were used. Results from at least three of the pure tone thresholds in decibel (dB) at 0.5–1–2–4 kHz had to be present to be analyzed as mean threshold [34]. The speech recognition tests were measured with a phonetically balanced (1) monosyllabic Norwegian word list specially made for children and with (2) three-word expressions (numeral + adjective + noun) [35]. The acoustical equipment was calibrated according to International Organization for Standardization [36,37] and followed recommended procedures [38,39]. Tympanometry (GSI Tympstar–Middle Ear Analyzer, Grason-Stadler Inc) was used to assess the status of middle ear functioning [40]. The results were categorized as either type A, B or C according to standard rules [41].

### Self-report questionnaire

The questionnaire included 16 questions, among them questions about subjective hearing and ear complaints, number of VT surgeries they had gone through, date of their most recent surgery, location and frequency of follow-ups after surgery, and eventual referral back to an otolaryngologist. Socio-demographic information included parental education and occupation. The questions had been pilot tested among employees at the Ear-Nose-Throat department before used in the study.

### Statistical methods

The groups were analyzed according to where the participants were scheduled to have follow-ups at time of surgery, not according to the guideline recommendations. Children scheduled for follow-ups by the outpatient clinic (n = 45) and by private otolaryngologists (n = 5) were analyzed as one group, the otolaryngologist group.

Data was read optically, quality assured and then analyzed with SPSS 21 and Stata 12. Categorical data were assessed with chi-square test and Stata Proportion test. Hearing thresholds and speech audiometry were not normally distributed, and therefore analyzed with non-parametric tests (Mann–Whitney and Hodges-Lehman tests). In addition, the results were retested with *t*-tests (using the assumption of a normally distributed mean) finding the same results as using the non-parametric tests. We present results from the *t*-tests; 95% CI was calculated from the difference of means between the groups. The differences in mean threshold and tympanometry during the follow-up period were analyzed for single ears that underwent VT surgery (excluding myringotomy only) and tested at both time points. Linear regression analysis of differences in hearing by type of follow up was performed adjusting for (1) age, (2) re-surgery and (3) shared care. This was done in separate analysis due to low statistical power. A sample size analysis showed that with a significance level of 0.05, power of 80% and a desire to show a 9 dB difference in mean threshold between the groups, 23 patients were needed in each group. As a result the present study includes enough patients to observe group differences in mean threshold of 9 dB or more.

## Results

A total of 89 children completed the audiological examination and 16 of these had severe medical syndromes. Of the 73 participants analyzed in this paper, 23 (31.5%) were scheduled for follow-ups by GPs and 50 (68.5%) by otolaryngologists. Two did not deliver the questionnaire. Not all participants had audiological tests before surgery (see Table 1), but no group differences were found. Those not hearing-tested were younger than those tested (2.5 vs 4.5 years, p < 0.01). There were no significant differences between the groups followed-up by GPs or otolaryngologists regarding socio-economic (age, gender, parental education) or audiological variables prior to surgery.

**Table 1:** Baseline characteristics of socio-demographic data and audiological measures by type of follow-up

	**Completed (n)**		**Type of follow-up:**		**∆ (95% CI)**
	**GP**	**Otolaryngologist**	**GP**	**Otolaryngologist**	
**Socio-Demographic data:**					
**Gender.** Female, n (%)	23	50	10 (43.5)	19 (38.0)	5.5% (-18.9, 29.8)
Male, n (%)			13 (56.5)	31 (62.0)	- 5.5% (-29.8, 18.9)
**Age at surgery.** Mean (min-max) years	23	50	3.4 (0.9-6.1)	3.9 (1.2-11.8)	˗ 0.5 yrs (-1.5, 0.6)
**Education.** One parent or more with higher education^1^, n (%)	23	47	20 (87.0)	36 (76.6)	10.4% (-8.0, 28.7)
**Audiological measures:**					
**Audiometry**^2^					
Mean threshold^3^ best ear, mean (SD) dB^4^	13	27	22.1 (10.0)	22.6 (13.7)	-0.5 dB (-9.2, 8.2)
Mean threshold worst ear, mean (SD) dB	12	27	32.8 (9.2)	33.1 (15.3)	-0.3 dB (-10.0, 9.4)
**Tympanometry**					
Effusion in one or both middle ears^5^, n (%)	12	30	11 (91.7)	26 (86.7)	5.0% (-14.8, 24.8)

^1^Higher education = education after completed high school ^2^Pure tone audiometry, play audiometry or informal hearing ^3^Mean Threshold (0.5–1–2–4 kHz) ^4^dB = decibel ^5^Tympanometry type B, not enlarged ear canal volume.

The results from the audiological data and the parental reports of child hearing and ear complaints two years after surgery are listed in Table 2. Some children underwent VT surgery again before the audiological examination in our study (see Table 2). The mean time since last surgery was thereby reduced, and was respectively 22 and 21 months.

**Table 2:** Audiological measures and parental report by type of follow-up 24 ± 3 months after surgery

	**Completed (n)**		**Type of follow-up**		**∆ (95% CI)**
	**GP**	**Otolaryngologist**	**GP**	**Otolaryngologist**	
**Audiometry**^**1**^					
Mean threshold^2^ best ear, mean (SD) dB^3^	22	50	11.7 (6.6)	16.2 (11.7)	-4.5 dB (-9.9, 0.8)
Mean threshold worst ear, mean (SD) dB	22	48	19.0 (11.2)	20.8 (14.0)	-1.9 dB (-8.6, 4.9)
**Speech recognition tests**					
**1. Three-words expression**^4^					
Best ear 50% perception, mean (SD) dB	16	33	17.0 (6.8)	20.7 (6.8)	-3.7 dB (-7.9, 0.5)
Worst ear 50% perception, mean (SD) dB	15	32	25.9 (13.3)	26.8 (12.8)	-0,9 dB (-9.0, 7.2)
**2 Monosyllabic words**					
Best ear max perception, mean (SD) dB	22	41	30.2 (7.5)	31.5 (6.1)	-1.2 dB (-4.7, 2.3)
Worst ear max perception, mean (SD) dB	22	40	37.7 (11.4)	37.4 (7.9)	0.5 dB (-4.6, 5.3)
**Tympanometry**					
Effusion in one or both middle ears^5^, n (%)	23	49	6 (26.1)	12 (24.5)	1.6% (-20.0, 23.2)
**Parental report of child hearing**^6^, n (%)					
Better	23	47	20 (87)	39 (83)	4.0% (-13.5, 21.4)
Unchanged			3 (13)	8 (17)	-4.0% (-21.4, 13.5)
Worse			0	0	0%
**Parental report of child’s ear complaints**^6^, n (%)					
Better	22	47	16 (72.7)	37 (78.7)	-6.0% (-28.0, 16.0)
Unchanged			5 (22.3)	9 (19.1)	3.2% (-17.2, 24.4)
Worse			1 (4.5)	1 (2.1)	2.4% (-7.2, 12.1)
**Re-surgery.** One or more surgery during the follow-up period, n (%)	23	50	6 (26.1)	13 (26.0)	0.1% (-21.6, 21.7)

^1^Pure tone audiometry, play audiometry or informal hearing tests ^2^Mean Threshold (0.5–1–2–4 kHz) ^3^dB = decibel ^4^Numeral + adjective + noun (see Methods) ^5^Tympanometry type B and not enlarged ear canal volume ^6^In comparison with before VT surgery.

The mean threshold for single ears (n_GP_ = 20 and n_otol_ = 39 ears) improved in both groups (both p values < 0.01) during the follow-up period. There were no significant differences in the mean hearing improvement between the GP and otolaryngologist groups (12.8 vs 12.6 dB, p = 0.9). The hearing improvement was still unaffected by scheduled groups of follow-up after adjusting for cofactors in separate analysis as age (p = 0.9), re-surgery (p = 0.9) and shared care (p = 0.7). The proportion of single middle-ears with effusion (n_GP_ = 20 and n_otol_ = 50 ears) was reduced in both groups after surgery (p < 0.01 in both groups). The GP group had a reduction from 90% (18/20) to 25% (5/20) giving a relative reduction of 78%, and the otolaryngologist group from 80% (40/50) to 20% (10/50), a relative reduction of 75%. There were no significant differences between the groups (p = 0.9).

In the questionnaire supplementary information about the follow-up care could be provided. Two participants feared lack of competence and equipment at the GP’s office; one was not satisfied with the otolaryngologist follow-ups and one commented lack of summoning by the otolaryngologist. Further data of user satisfaction was not conducted. This study has not assessed other complications than reduced hearing and middle ear function.

## Discussion

Implementation of new clinical guidelines for follow-ups after insertion of VTs did not negatively affect audiological outcomes or subjective hearing complaints two years after surgery. Regardless of whether the follow-ups were done by GPs or by otolaryngologists, we found improved mean hearing thresholds (12.8 dB vs 12.6 dB) and a reduced percentage of middle ears with effusion (78% vs 75%).

The strength of this study was that the participants were tested with pure tone audiometry, speech audiometry and tympanometry which give a better overall view of the audiological status than just pure tone audiometry. However, nearly 40% of the children did not have a formal audiological evaluation before surgery because of their low age and difficulties in getting them to cooperate in the tests. The low number of participants implies a possibility that the material lacks power to detect important clinical differences; i.e. type 2 errors. Still, the differences we observed between the groups in mean threshold (0.2 dB) and tympanometry (3%) were so small that if they represent the true values, the differences between the groups are not clinically relevant.

The best method for research on comparing groups is a randomized controlled design with the purpose of giving valid information about the chosen method’s efficacy. This was not done in our study. However, the two study groups in our material did not differ by age, sex, parental education or audiological evaluation prior to surgery, even though the otolaryngologists were meant to follow up those with the worst hearing. This was surprising. An explanation could be that the otolaryngologist after a clinical examination considered the location of follow-ups differently than the guidelines, but it is also possible that the guidelines were not precise enough to allocate follow-ups. Again, there is a possibility of type 2 errors. There was a difference though in number of participants in the groups (23 in the GP group vs 50 in the otolaryngologist group). Nevertheless, the aim of this study has not been to measure the “best follow-up”, but to examine if follow-up care by the GP can be done without increasing the risk of harm.

### Audiological outcome

An increasing number of studies, including the previous mentioned Cochrane report [6], have concluded that there is little or no long-term hearing effect of VT surgery [42,43]. This challenges the need for all children to be controlled by an otolaryngologist, i.e. at a more expensive healthcare level than primary care. In contrast to the Cochrane report, our study demonstrated improved hearing and better middle ear function two years after surgery. Our material was small, and one-fourth of the patients had undergone another surgery in the follow-up period. Also, the interpretation of effusion in the middle ear is difficult because of the possibility of intercurrent disease giving effusion for a short period. This implicates that the results should be interpreted carefully. However, despite adjustment for re-surgery, age and shared care, the improvement of the hearing thresholds and middle ear function were not affected by the group of physicians doing the follow-ups. As far as we know, very few studies have investigated differences in audiological outcome by the follow-up strategy.

### Handling complications

Controls after VT surgery are practiced differently internationally, and as the Swedish SBU concluded there is no evidence that one way is superior to another [16]. Thus, once surgery has been performed, it is important to control for complications and to follow up the disease that led to surgery [8]. Some claim that delegating controls to the GPs may lead to increased complications or risk of overlooking a sensorineural hearing loss because they lack experience and good enough equipment to control the children; for instance do very few have otomicroscopy or audiometry [13]. This concern was also mentioned by two of the participants. Severe complications are however rare [44]. According to a meta-analysis “sequelae of tympanostomy tubes are common but are generally transient (otorhea) or cosmetic (tympanosclerosis, focal atrophy)” [9]. The GPs were given a guideline that included advise about how to handle some complications [18]. But still it is possible that these, and other complications, may not be handled according to best practice. However, the GPs can refer back if he or she is uncertain about how to handle complications. In our material 60% were referred back [33]. Reasons for referral back were not assessed, but we discovered that about one-fourth had new ventilation tubes in the follow-up period, so recurrent disease seems to be one reason.

### Accessibility

In Norway, the population needs referral from a GP to get access to the public specialist health care system. A list-based system in primary care was established in 2001. As a result, nearly the entire population has one specific GP to consult. This makes it easier to get a consultation with a GP than an otolaryngologist. The accessibility in general practice is also better if the child needs help at another point of time than the specified controls six and 18 months after surgery; for instance because of suspected complications, reduced hearing or questions after surgery. We have earlier documented that one-third of the children went to the GP to control the VTs even though they were scheduled for follow-ups only at the outpatient clinic [33]. This indicates that some degree of shared care will occur. When it comes to diseases like otitis media with effusion or recurrent otitis media with various complaints and need for treatment, the flexibility of follow-ups and shared care may be regarded as an advantage for the patients and their parents.

### Future research

Further studies are needed before implications for follow-ups after VT surgery are taken into consideration. A power estimated randomized controlled trial is recommended in order to explore differences in change of hearing thresholds, middle ear function, subjective complaints and complications by type of follow-ups. Future studies should also consider including user satisfaction and other aspects related to the quality of control.

## Conclusion

Implementation of new clinical guidelines for follow-ups after insertion of VTs, allowing GPs to control the VTs in one group of children, did not negatively affect audiological outcomes two years after surgery. Regardless of whether the follow-ups were done by GPs or otolaryngologists we found improved hearing thresholds and reduced amount of middle ears with effusion. No differences were found in the parental report of the child’s subjective hearing or ear complaints. Because of the limited size of the material we cannot exclude the possibility of overseeing small differences among the two groups. Complications and user satisfaction have not been assessed. Further research is needed to consider the implications for follow-ups after VT surgery.

## Abbreviations

VT: Ventilation tube; GP: General practitioner; dB: Decibel.

## Competing interests

The authors declare that they have no competing interests.

## Authors’ contributions

BA participated in design of the study, analysis and interpretation of the data and drafted the manuscript. VB and ASH participated in the design of the study, supervising and editing the manuscript; IH participated in supervising and editing the manuscript. AHO and SW contributed in data collection. All authors read and approved the final manuscript.

## Authors’ information

BA is specialist in general practice and a research fellow.

IH is specialist in general practice and professor.

VB is an otolaryngologist and associate professor.

AHO and SW are audiologists.

ASH is RN and researcher.

## Pre-publication history

The pre-publication history for this paper can be accessed here:

http://www.biomedcentral.com/1472-6815/14/2/prepub
